# Mrs. R. G. Burden

**Published:** 1939

**Authors:** 


### MRS. R. G. BURDEN.

*Warden of Stoke Park Colony, Stapleton.*

On 19th September at Clevedon Hall, Somerset, Mrs. R. G. Burden, Warden of Stoke Park Colony, passed away after a long illness patiently and bravely borne. Her benevolence was generous and widely distributed, but in no direction was it more lavishly displayed than in her endowments for research into mental disorders. In 1933 she established the Burden Mental Research Trust with a gift of £10,000 for the encouragement and prosecution of research into mental problems and disorders under the direction of Professor Berry and under the ægis of the British Medical Association. In May, 1939, she established the Burden Neurological Research Institute with Professor F. L. Golla as Director. Mrs. Burden was the second wife of the late Rev. H. N. Burden, whose life-work on behalf of the feeble-minded can never be forgotten.

As Miss R. G. Williams she had been invited to help in the organization of Stoke Park Colony at its foundation in 1913 ; but the temporary help which she originally came to give led to her becoming the Superintendent of the Colony, an office which she continued to fill with wholehearted devotion to the end of her life. For even after her marriage to the first Warden, Rev. H. N. Burden, she discharged the duties of superintendent, and on his death she succeeded him as warden and combined the two offices. By her self-sacrificing support of her husband's interest in mental deficiency during his lifetime and her intense loyalty to his ideals after his death Mrs. Burden won the admiration of all who knew her. The impelling force and example of the Burdens is the principal factor which has led to the present provision of institutions for the care of mental defectives throughout the country.

Their persistence, their wise use of their great wealth, and their indifference to official coldness or public apathy, led at long last to official imitation of their projects.

They carried out undeviatingly the advice of Thomas à Kempis to seek one good, one end, so zealously that nothing else could come into competition or partnership with it.

No other memorial of them could surpass the great institutions they founded and fostered.

